# Uncommon manifestations of a rare disease: a case of autoimmune GFAP astrocytopathy

**DOI:** 10.1186/s12883-021-02070-6

**Published:** 2021-01-27

**Authors:** Hong Di, Yue Yin, Ruxuan Chen, Yun Zhang, Jun Ni, Xuejun Zeng

**Affiliations:** 1 Department of General Internal Medicine, Peking Union Medical College Hospital, Chinese Academy of Medical Science and Peking Union Medical College, Beijing, 100730 China; 2 Department of Internal Medicine, Peking Union Medical College Hospital, Chinese Academy of Medical Science and Peking Union Medical College, Beijing, 100730 China; 3 Department of Neurology, Peking Union Medical College Hospital, Chinese Academy of Medical Science and Peking Union Medical College, Beijing, 100730 China

**Keywords:** Glial fibrillary acidic protein (GFAP) astrocytopathy, Hyponatremia, Hypokalemia, Fatigue

## Abstract

**Background:**

Manifestations of intractable hyponatremia and hypokalemia in autoimmune glial fibrillary acidic protein (GFAP) astrocytopathy have been rarely reported.

**Case presentation:**

A 75-year-old male patient presented as the case of syndrome of inappropriate antidiuretic hormone secretion (SIADH) and intractable hypokalemia, showed fever, fatigue, and mental disorders. Signs and symptoms of meningoencephalitis, ataxia, and cognitive abnormalities. Magnetic resonance imaging (MRI) revealed multiple white matter lesions of the central nervous system. He had GFAP-IgG in the cerebrospinal fluid (CSF). After treatment with corticosteroids, his symptoms were alleviated gradually, and the level of electrolytes was normal. However, head contrast-enhanced MRI + susceptibility-weighted imaging (SWI) showed a wide afflicted region, and the serum GFAP-IgG turned positive. Considering the relapse of the disease, ha was treated with immunoglobulin and mycophenolate mofetil (MMF) to stabilize his condition.

**Conclusion:**

This case showed a rare disease with uncommon manifestations, suggesting that careful examination and timely diagnosis are essential for disease management and satisfactory prognosis.

## Background

Autoimmune GFAP astrocytopathy is a new autoimmune disease of the central nervous system (CNS). The common clinical features, including fever, headache, encephalopathy, involuntary movement, myelitis, and visual abnormalities, have been reported. Antibodies in CSF against GFAP-α is a biomarker and expressed in most cases with autoimmune GFAP astrocytopathy. Furthermore, the clinical manifestations were mostly typical, and obvious electrolyte disorder was rarely reported in this disease. Although only 9 cases of hyponatremia in autoimmune GFAP astrocytopathy have been reported, all the cases showed hyponatremia during hospitalization that was not presented as the initial symptom. Herein, we firstly reported a case of autoimmune GFAP astrocytopathy presented as SIADH and intractable hypokalemia at the onset of the disease.

## Case presentation

A 75-year-old male, with a history of hypertension, coronary heart disease and atrial fibrillation, presented at our hospital (Peking Union Medical College Hospital, Beijing, China) on May 23, 2019, with fever, anorexia, and fatigue for > 1 month. Since April 22, the patient had developed a recurrent fever for unknown causes, accompanied by anorexia and fatigue. His temperature peaked twice daily at a maximum of 38.5 °C. The symptoms worsened gradually after 10 days, and at a community hospital, high white blood cells and low blood sodium were detected. He was administered electrolyte supplements and cephalosporins for 3 days. The fever was controlled, hyponatremia was not corrected, and anorexia and fatigue were aggravated. Slowly, he was unable to walk without assistance. On May 16, the patient developed unresponsiveness, lethargy, and dysuria, and was sent to several hospitals afterward. Physical examination revealed that the tendon reflexes of the lower limbs were weakened, and Romberg’s sign was positive;cranial nerve function,sensory function, muscle force and tension were all normal,bilateral Babinski signs and Kernig’s sign were negative. Laboratory tests showed that the white blood cell count fluctuated between 8.49 and 14.85 × 10^9^/L (predominantly neutrophils), and serum electrolyte levels were 128 mmol/L for sodium (Na^+^), 86 mmol/L for chloride (Cl^−^), and 3.2 mmol/L for potassium (K^+^). Other liver and kidney indexes, ESR, CRP, immunoglobulin, complement, myocardial enzymes, and BNP were unremarkable. The 24-h Holter test revealed sustained atrial fibrillation. Lung computed tomography (CT) showed bronchiectasis with slight inflammatory exudation and emphysema. Brain MRI revealed multiple abnormal signals in the brain stem, the bilateral basal ganglia, the thalamus, the corpus callosum, the left frontal lobe, and the left semi-oval center, leading to a preliminary diagnosis of pulmonary infection with cerebral lacunar infarction. The patient was then catheterized and given sodium supplements and ceftazidime for 7 days, followed by levofloxacin for 3 days and low molecular weight heparin 5000 U once daily. Consequently, there was no visible improvement, and the patient was transferred to our hospital for further investigation and treatment on May 27. The patient had been in a feeble condition during the disease course. He had poor sleep and reduced appetite and had lost 15 kg in weight. During hospitalization at other hospitals, he had a history of nausea and vomiting. He also had experienced diarrhea but defecated only once or twice a day. Nonetheless, his urine volume and color were normal. On admission, the patient was afebrile but still unconscious. He had a blood pressure of 114/86 mmHg and a heart rate of 103 beats/minute. His body mass index (BMI) was 19.4 kg/m^2^. The superficial lymph nodes were not palpable. No rales or rhonchi were heard. Arrhythmia, short pulse, and cardiac auscultation were generally normal. The liver and spleen were not palpable below the ribs. Also, no limb edema was detected. Ataxia and suspicious neck resistance were noted. He had normal muscle tension, knee reflexes were elicited, and negative Babinski signs were observed. The patient was unable to cooperate for further examination.

The patient’s blood electrolytes test documented that the Na^+^ was 129 mmol/L, and K^+^ was 3.1 mmol/L; therefore, sodium and potassium were supplemented. Blood and urine osmotic pressure was normal. Thyroid and adrenal functions were also normal. Therefore, the SIADH was suspected and potential causes were investigated. The following tests were either normal or negative: anti-HCV, anti-HIV, and anti-syphilis antibodies; *Legionella pneumophila* antibodies; blood *Cryptococcus* antigen (qualitative) test, and *Brucella* agglutination test; T-spot. TB test; inflammatory markers; anti-nuclear antibodies; Lupus anticoagulant; antiphospholipid antibodies, anti-neutrophil cytoplasmic antibodies; blood tumor biomarkers and serum immunofixation electrophoresis; positron emission tomography/computed tomography (PET/CT).

**Fig. 1:**
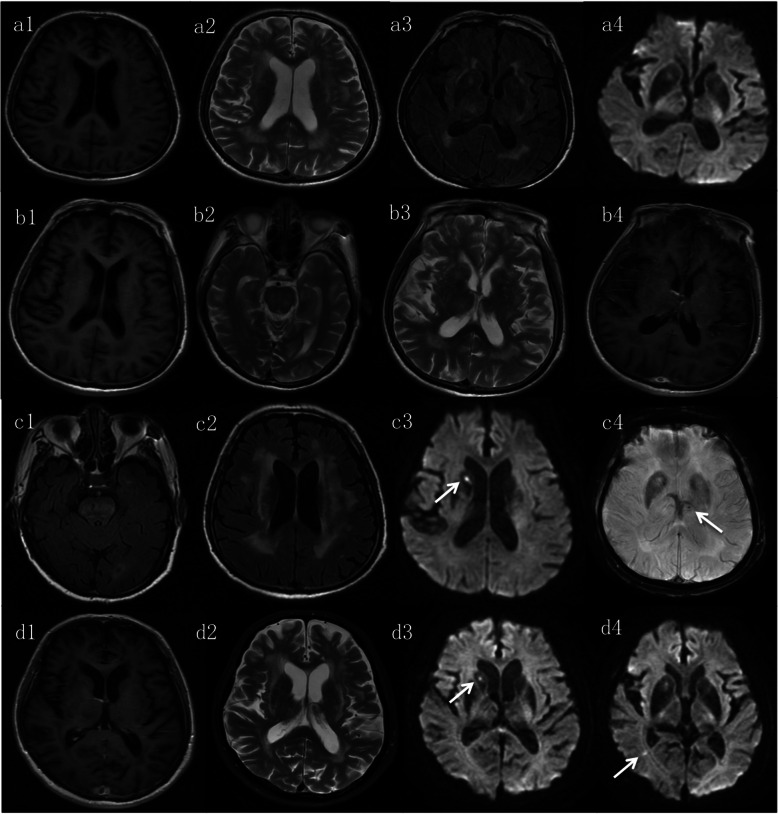
MRI findings of the patient. MRI on May 31, 2019 (a1–a4) showed patchy abnormal lesions in the bilateral thalamus, basal ganglia, pons, and bilateral white matter on T2 and FLAIR image. MRI with contrast on June 5 (b1–b4) revealed no enhanced lesions on the T1 image and the abnormal lesions on the T2 image were similar to the previous MRI scan. On July 26 (c1–c4), the FLAIR images revealed that abnormal signals were more severe and diffused than the previous MRI findings. DWI image showed new cerebral infarction in the right basal ganglia, and SWI found a small hemorrhage in the left thalamus (indicated by arrows). MRI with contrast on July 30 (d1–d4), new cerebral infarction was found in the right optic radiation, and the infarction of the right basal ganglia was similar to the previous scan (indicated by arrows). Also, no lesions were enhanced. DWI, diffusion-weighted imaging; FLAIR, fluid-attenuated inversion recovery; MRI, magnetic resonance imaging; SWI, susceptibility-weighted imaging

Lumbar puncture revealed normal opening pressure. The total white blood cell count of the CSF was 34 × 10^6^/L, consisting of all mononuclear cells (LY% 90, MONO% 10). Total protein was 1.58 g/L, and glucose was not decreased. IL-6 and TNF-α were elevated, while IL-8 and IL-10 were normal. Epstein–Barr virus (EBV)-DNA was 500 copies/mL, and Cytomegalovirus (CMV)-DNA and next-generation sequencing (NGS) were unremarkable. Anti-LGI1, anti-CASPR2, anti-NMDA and Hu.Yo.Ri antibodies in the blood and the CSF were negative. Brain contrast-enhanced MRI + diffusion-weighted MRI (DWI) + fluid-attenuated inversion recovery (FLAIR) + SWI displayed multiple abnormal signals in both thalami, the basal ganglia, the left cerebellum, the pons, and the white matter of the bilateral cerebrum (Fig. 1a1-a4, b1-b4).

After excluding the potential infections or malignancies, multiple autoimmune leukodystrophy of the nervous system was considered, and since June 18, after explaining the current condition to the patient, he agreed to be on intravenous infusion of immunoglobulin (IVIG) 20 g daily for 5 days and intravenous methylprednisolone 40 mg daily. The patient began to gain strength in the legs on day 3 and could stand with assistance. On June 25, using the tissue based indirect immune-fluorescence test, we detected that anti-GFAP antibody was positive for CSF, while anti-GFAP and anti-MOG antibodies for serum were negative, thereby leading to the diagnosis of autoimmune GFAP astrocytopathy. The patient was given intravenous methylprednisolone 1 g daily for 3 days, followed by an oral taper with prednisolone 50 mg daily. After the first 3 days, his blood sodium was maintained with oral supplements. The patient was conscious and had good orientation, and thus, was therefore discharged on June 28.

The patient’s symptoms improved gradually, and hence, the use of prednisolone was reduced. The patient was on 40 mg daily prednisolone during the subsequent follow-up and no sodium supplements; his blood electrolytes were within the normal range (K^+^ 4.2 mmol/L and Na^+^ 141 mmol/L). CSF analysis revealed that white cell count dropped to 22 × 10^6^/L, and the protein level was lowered to 0.67 g/L. Nonetheless, on August 9, anti-GFAP antibody in CSF and serum were both positive on a repeat testing, and head contrast-enhanced MRI + SWI showed a wide afflicted region (Fig. 1c1-c4, d1-d4). Given that the disease was not well-controlled, 20 g daily intravenous immunoglobulin was administered for 5 days along with 0.5 g MMF twice daily. Consequently, the patient reported improved strength of the upper and lower limbs and could walk for 2 km on plain ground. In the subsequent follow-ups, he was on a continual gradual reduction in prednisone that was finally maintained at a 5 mg daily dosage plus 0.5 g MMF twice/day.

## Discussion and conclusions

This is a case of autoimmune GFAP astrocytes disease presented as SIADH and intractable hypokalemia. Fever, fatigue, and mental disorders were prominent in this patient. Severe electrolyte disorders could lead to similar clinical manifestations but not explain the signs of meningoencephalitis, ataxia, and cognitive abnormalities. Further examinations showed an increased CSF lymphocyte count and protein level and multiple white matter lesions of the central nervous system, accompanied by positive CSF anti-GFAP antibodies. These manifestations pointed to a diagnosis of GFAP astrocytopathy.

GFAP astrocytopathy is a newly defined autoimmune disease of the central nervous system. It usually occurs in patients > 40 (median age of onset: 44–50)-years-old [1] and affects men and women equally. A study of 19 Chinese GFAP patients showed that females accounted for 68%(13/19) of the total cases, and the overall median age of onset was 54 (23–73) years. Among the 102 GFAP patients reported by the Mayo clinic, 54%(55/102) were female, and the overall median age of onset was 44 (8–102)-years-old [2]. The disease is acute or subacute, assuming a gradually aggravating or relapsing-remitting pattern. The clinical manifestations include fever, headache, encephalopathy, involuntary movement, myelitis, and visual abnormalities. The study by Lennon et al., comprising of 102 patients [3] found that the three most common clinical manifestations were encephalitis and meningitis (54.5%, 56/102), myelitis (10.5%, 11/102), and encephalomyelitis (8%, 8/102). However, a Chinese study on 19 patients with GFAP astrocytopathy disease showed [4] that myelitis (68.4%), headache (63.2%), and optic neuritis (63.2%) were common manifestations. Currently, there is no diagnostic consensus for GFAP astrocytopathy. Accumulating evidence including positive anti-GFAP-IgG antibodies in the CSF, signs of the central nervous system inflammation, or specific manifestations on the cranial MRI support the clinical suspicion of GFAP astrocytopathy. Among these, the anti-GFAP antibodies in the CSF, especially at a higher titer than that in the serum, confirmed the diagnosis. However, for patients with typical presentations of meningoencephalomyelitis, whose anti-GFAP antibodies are negative, clinical judgment should be made based on a careful appraisal of other evidence [2]. Remarkable linear enhancement oriented radially to the ventricles in cranial MRI, which is also a specific manifestation of this disease. Reportedly [1], that normal cranial and spinal MRI is rare in patients with GFAP astrocytopathy, and the T2-weighted sequences would reveal some abnormalities in about half of the patients. In this case, the patient’s clinical manifestations are consistent with the common clinical manifestations of the disease. However, the patient’s condition was complicated by prominent electrolyte disorders. Hyponatremia was considered to be caused by SIADH. Thus, it could be speculated that hyponatremia might be caused by excessive production of antidiuretic hormone (ADH) as a result of the lesions in the hypothalamus. The electrolyte disturbances were controlled after treatment with steroids, which confirmed that SIADH was secondary to GFAP astrocytopathy. The study by Kimura et al. encompassed 14 patients with GFAP astrocytopathy disease [5]; 8/14 (57%) showed hyponatremia during hospitalization. A retrospective study of 19 patients with GFAP astrocytopathy in China [4] showed that only 1 patient presented SIADH.

Currently, the pathophysiological mechanism of GFAP astrocytopathy is not clarified. In a previous pathological study on GFAP astrocytopathy, MRI located lesions near the small blood vessels, with fewer astrocytes and showing dysregulation of T cells. Previous case reports [1] showed that GFAP astrocytopathy might be secondary to other diseases. About 25% of the patients were found to have tumors, among which, 75% were teratomas, and 20% of the patients had autoimmune diseases, including type 1 diabetes and rheumatoid arthritis. Another study [4] showed that 13/19 Chinese patients had other autoantibodies, including anti-nuclear antibody, anti-endothelial cell antibody, anti-neutrophil cytoplasm antibody, anti-double-stranded DNA antibody, anti-RA33 antibody, and anti-SSA/Ro antibodies. About 30–34% of the cases were preceded by an infection. Our case is an elderly male. Although there is no evidence of malignancies, the disease could still be a sign of the paraneoplastic syndrome before the tumor became visible. Therefore, regular tumor screening should be considered. Moreover, no evidence of autoimmune diseases was found. The infection screening found that EBV-DNA was low titer but positive in cerebrospinal fluid; however, the patient had no manifestations of the infection. Also, the high sensitivity second-generation sequencing to evaluate the CSF pathogenesis did not find any evidence of viral infection; therefore, EBV infection was not considered.

The hallmark of autoimmune GFAP astrocytopathy is immunosuppressive therapy. Typically, the disease has good response to the initial treatment with glucocorticoids in a monophasic course. However, about 20% of the patients experience disease relapse or become steroid-dependent and require combined immunosuppressive therapy [1]. At present, the reported prognosis of the disease is diversified. Some patients reported a good prognosis, while the long-term follow-up of 7 patients in China [6] showed that severe disability and poor prognosis are common, which might be related to delayed diagnosis (the average time for diagnosis is 12 months) and severe symptoms. Our patient was diagnosed 1 month after the disease onset, which was significantly shorter than the average. Interestingly, symptoms abated after treatment with steroids, yet the continuously positive antibodies and the progression in imaging warranted combined immunosuppressant therapy. Moreover, clinicians should be cautious while monitoring potential malignancies or other underlying processes during follow-ups.

Electrolyte disorders are not uncommon in the elderly but occasionally could reveal severe diseases. Thus, careful examination and increased awareness of rare etiologies  are required in clinical decision-making for optimal disease management and improved prognosis.

## Data Availability

All data generated and analysed during this study are included in this article.

## Abbreviations

GFAP: Glial fibrillary acidic protein
SIADH: Syndrome of inappropriate antidiuretic hormone secretion
MRI: Magnetic resonance imaging
CSF: Cerebrospinal fluid
SWI: Susceptibility-weighted imaging
MMF: Mycophenolate mofetil
CNS: Central nervous system
CT: Computed tomography
BMI: Body mass index
PET/CT: Positron emission tomography/computed tomography
EBV: Epstein–Barr virus
CMV: Cytomegalovirus
NGS: Next-generation sequencing
DWI: Diffusion-weighted MRI
FLAIR: Fluid-attenuated inversion recovery
ADH: Antidiuretic hormone
